# Differences in endothelial function between patients with Type 1 and Type 2 diabetes: effects of red blood cells and arginase

**DOI:** 10.1042/CS20240447

**Published:** 2024-08-05

**Authors:** John Tengbom, Eftychia Kontidou, Aida Collado, Jiangning Yang, Michael Alvarsson, Jonas Brinck, Sophia Rössner, Zhichao Zhou, John Pernow, Ali Mahdi

**Affiliations:** 1 Division of Cardiology, Department of Medicine Solna, Karolinska Institutet, Karolinska University Hospital, Stockholm, Sweden; 2 Division of Endocrinology and Diabetology, Department of Molecular Medicine and Surgery, Karolinska Institutet, Karolinska University Hospital, Stockholm, Sweden; 3 Division of Endocrinology, Department of Medicine Huddinge, Karolinska Institutet, Karolinska University Hospital, Stockholm, Sweden

**Keywords:** arginase, diabetes mellitus, endothelial dysfunction, red blood cells

## Abstract

The mechanisms underlying endothelial dysfunction in Type 1 and Type 2 diabetes (T1DM and T2DM) are unresolved. The red blood cells (RBCs) with increased arginase activity induce endothelial dysfunction in T2DM, but the implications of RBCs and the role of arginase inhibition in T1DM are unexplored. We aimed to investigate the differences in endothelial function in patients with T1DM and T2DM, with focus on RBCs and arginase. Thirteen patients with T1DM and twenty-six patients with T2DM, matched for HbA1c and sex were included. *In vivo* endothelium-dependent and -independent vasodilation (EDV and EIDV) were assessed by venous occlusion plethysmography before and after administration of an arginase inhibitor. RBCs were co-incubated with rat aortic segments for 18h followed by evaluation of endothelium-dependent (EDR) and -independent relaxation (EIDR) in isolated organ chambers. *In vivo* EDV, but not EIDV, was significantly impaired in patients with T2DM compared with patients with T1DM. Arginase inhibition resulted in improved EDV only in T2DM. RBCs from patients with T2DM induced impaired EDR but not EIDR in isolated aortic segments, whereas RBCs from patients with T1DM did not affect EDR nor EIDR. The present study demonstrates markedly impaired EDV in patients with T2DM in comparison with T1DM. In addition, it highlights the divergent roles of RBCs and arginase in mediating endothelial dysfunction in T1DM and T2DM. While endothelial dysfunction is mediated via RBCs and arginase in T2DM, these phenomena are not prominent in T1DM thereby indicating distinct differences in underlying mechanisms.

## Introduction

Type 1 and type 2 diabetes mellitus (T1DM and T2DM) both represent major clinical burdens associated with hyperglycemia but with different underlying pathophysiology [1]. Both groups of patients have severely increased risk of developing macrovascular complications including myocardial infarction and stroke [2]. While both patient groups have increased risk of microvascular complications such as retinopathy and nephropathy, the prevalence of these complications is higher among patients with T1DM compared with patients with T2DM [3,4]. T1DM is considered an autoimmune disease with autoantibodies leading to destruction of beta cells, resulting in insulin deficiency with normal body mass index and normal triglyceride levels at diagnosis. On the other hand, T2DM develops due to lifestyle factors and is largely part of the metabolic syndrome, including insulin resistance, increased body mass index, and elevated triglyceride levels [5]. Although both types of diabetes result in hyperglycemia and oxidative stress, there are numerous differences in the mechanisms by which these events are orchestrated. For instance, insulin deficiency is present at disease onset in T1DM, whereas insulin is elevated in the early phase of T2DM as a result of insulin resistance. Due to these distinct underlying disease mechanisms, clear differences exist between T1DM and T2DM regarding hyperglycemia, insulin levels and oxidative stress [6,7]. Hyperglycemia has long been considered to be a trigger of diabetic complications, but large clinical trials aiming at reducing cardiovascular complications in patients with T2DM by intensified glycemic control have shown conflicting results [8,9]. However, available data suggest that improvement in glycemic control delays the onset of microvascular complications [9,10]. It is believed that hyperglycemia represents a major driver behind early changes in the microvasculature by causing a shift in the redox balance toward increased oxidative stress and reduced bioavailability of nitric oxide (NO) in endothelial cells [11]. This endothelial dysfunction is an early hallmark of injury in the vascular wall and a prerequisite for the initiation of atherosclerotic cardiovascular disease.

Endothelial dysfunction in diabetes involves, among several changes, upregulation of arginase in endothelial cells [12,13]. This enzyme is an important regulator of NO bioavailability by its reciprocal inhibition of NO formation by utilizing the same substrate, L-arginine, as the NO-producing enzyme endothelial NO synthase (eNOS). Increased arginase activity is not only associated with reduced NO production but also with increased formation of reactive oxygen species (ROS) [14]. In two studies, with 48 and 24 subjects included, forearm endothelial function was improved following intra-arterial administration of an arginase inhibitor [15,16], indicating the involvement of arginase in endothelial dysfunction *in vivo*. Interestingly, this effect was obtained irrespective of glycemic control [17], suggesting that arginase inhibition may exert beneficial effects on vascular function beyond improved glucose control in T2DM. However, the role of arginase in endothelial dysfunction in patients with T1DM is unknown.

Red blood cells (RBCs) have gained increasing attention as it has been shown that RBCs play a major role in the pathophysiology of vascular injury in T2DM [18]. We have previously shown that RBCs induce endothelial dysfunction in patients with T2DM via a mechanism that involves up-regulation of vascular arginase activity and ROS formation [19]. Furthermore, pharmacological inhibition of arginase prevented the RBC-induced endothelial dysfunction [19], supporting a key functional role of RBCs in T2DM. Whether RBCs mediate endothelial dysfunction in T1DM is unknown, however.

Taken together, available data give strong support for the presence of endothelial dysfunction in patients with T2DM. However, evidence concerning endothelial dysfunction in patients with T1DM is scarce and more importantly, no previous study has compared the two diseases matched for glycemic status with regards to the difference in *in vivo* endothelial function. We therefore aimed to investigate the difference in endothelial function in patients with T1DM and T2DM and the signaling behind these effects with focus on arginase and RBCs.

## Methods

### Subjects

Patients with T1DM (*n*=13) and patients with T2DM (*n*=26), ≥ 40 years of age were recruited from the Department of Endocrinology, Karolinska University Hospital, Stockholm, Sweden and the Center for Diabetes, Academic Specialist Center, Health Care Services of the Stockholm County, Sweden. T2DM was defined according to the criteria outlined by the World Health Organization [20]. Patients in the two groups were matched for HbA1c. Exclusion criteria were: acute coronary syndrome or stroke within 6 months prior to inclusion, ongoing treatment with oral anticoagulants or history of Raynaud’s phenomenon. The patients were investigated after an overnight fasting period and were instructed to take half their normal dose of insulin the evening before the investigation or blood sampling. On the day of the sampling or experiments, the patients did not take any morning medication, including insulin. Routine blood chemistry parameters were analyzed at the Karolinska University Hospital Laboratory. Insulin was quantified in plasma using a Human Insulin ELISA kit (ThermoFisher Scientific, Waltham, MA, U.S.A., Cat no; KAQ1251) according to the manufacturer’s instructions.

The data from forearm blood flow evaluations of patients with T2DM (*n*=12) were included from a previous study [17] but used in another context in the present study.

### Venous forearm occlusion plethysmography

All experiments were carried out with patients in the supine position in a temperature-controlled environment following a light standardized breakfast after an overnight fasting period. To evaluate *in vivo* endothelium-dependent vasodilatation (EDV) and endothelium-independent vasodilatation (EIDV), venous forearm occlusion plethysmography was used [15,17]. In short, the brachial artery of the non-dominant arm was cannulated under sterile conditions. Forearm blood flow (FBF) was determined by obstructing the venous outflow from the forearm by inflating a cuff placed around the upper arm to supra-venous pressure (50 mmHg) in repeated cycles of 10s separated by 10 s of deflation. The changes in forearm circumference were registered by an indium-gallium strain gauge. The circulation in the hand was excluded by inflation of a wrist cuff to 30 mmHg above the systolic blood pressure. After a period of stabilization, baseline FBF was measured under constant infusion of 0.9% NaCl. To assess EDV and EIDV, serotonin (21, 70 and 210 ng/min) and sodium nitroprusside (SNP; 1, 3 and 10 µg/min) respectively, were infused via the arterial cannula. To investigate the effect of arginase inhibition on vascular function, EDV and EIDV were re-assessed following 120 min intra-arterial infusion of the arginase inhibitor N^ω^-hydroxy-nor-L-arginine (nor-NOHA; 0.1 mg/min). All doses were based on previous studies performed by the group [15].

### RBC isolation

Whole blood from patients with T1DM and T2DM were collected in heparin tubes and kept on ice before centrifugation at 4°C and 1000 ***g*** for 10 min, after which the plasma and buffy coat were discarded. The RBCs were then washed for three cycles with Krebs-Henseleit (KH) buffer and centrifuged at 4°C and 1000 ***g*** for five min. This protocol results in the removal of >99% of leucocytes and 98% of platelets [19,21].

### Rat aortic segments preparation

Male Wistar rats (Charles River Laboratories, Sulzfeld, Germany) at the age of 10–15 weeks were anesthetized with intra-peritoneal injection of pentobarbital sodium (50 mg/kg). When anesthetized, thoracotomy was performed, and the thoracic aorta was carefully extracted and kept in KH buffer on ice. Connective tissue and perivascular adipose tissue were then removed gently from the aortic rings before being used in *ex vivo* experiments as described below.

### Isolated organ chamber experiments

Isolated RBCs were diluted to a hematocrit of ∼45% with KH buffer and then incubated with rat aortic segments at 37°C and 5% CO_2_ for 18 h. After incubation, the aortic segments were gently rinsed with KH buffer and then evaluated for endothelium-dependent and -independent relaxations (EDR and EIDR, respectively) in an organ chamber system (Danish Myo Technologies A/S, Hinnerup, Denmark). During the organ chamber experiments, the aortic segments were submerged in KH buffer and bubbled with 5% CO_2_ and 95% O_2_ at a constant temperature of 37°C. All vessels were preconstricted with phenylephrine (10^−7^ M to 10^−6^ M). Relaxation was achieved with cumulatively increasing concentrations of acetylcholine (10^−9^ M to 10^−5^ M) to determine EDR followed by a single concentration of SNP (10^−5^ M) to determine EIDR.

### Statistics

All data are presented as mean ± SEM unless otherwise stated. Normality was tested with D’Agostino-Pearson’s normality test. Data from the venous occlusion forearm plethysmography are presented as the change in blood flow from baseline (during saline infusion) during infusion of serotonin and SNP and are expressed as ml/min/1000 ml. Data from the myograph chamber experiments are expressed as percent relaxation from the preconstriction tone by each concentration of acetylcholine or a single concentration of SNP. Statistical differences for dose-response curves were analyzed with two-way ANOVA with repeated measures. Categorical data were compared using Fischer’s exact test. Non-categorical data were compared using Student’s *t*-test, Mann–Whitney *U*-test or Wilcoxon matched pairs signed rank test depending on distribution and pairing. Statistical significance was accepted when *P<*0.05.

## Results

Characteristics and medication of the included patients are presented in Table 1. As a result of effective matching, the groups did not differ in HbA1c. The patients with T2DM were slightly older, had higher body mass index and plasma triglycerides compared with the patients with T1DM. No major differences were observed in RBC indices. T2DM patients had severe insulin resistance reflected by the high Homeostatic Model Assessment for Insulin Resistance (HOMA-IR) index. Furthermore, they were more frequently treated with cardiovascular protective drugs, including angiotensin-converting enzyme or angiotensin receptor blockers and aspirin. As expected, the groups differed in type of diabetes medication. The patients with T1DM had a longer duration of diabetes and higher high-density lipoprotein cholesterol levels.

**Table 1:** Baseline characteristics of study subjects

	T1DM (*n*=13)	T2DM (*n*=26)
Age, years	60 ± 10	67 ± 8 *
Sex, female:male, *n* (%)	5:8 (38:62)	7:19 (27:73)
Duration of diabetes, years	28 ± 17	13 ± 7 *
BMI, kg/m^2^	26 ± 5	31 ± 4**
Systolic BP, mmHg	137 ± 9	140 ± 16
Diastolic BP, mmHg	82 ± 6	78 ± 8
Smoking, *n* (%)	0 (0)	4 (15)
Fasting glucose, mM	11.2 ± 2.9	10.8 ± 3.4
HbA1c, mmol/mol	67 ± 8	71 ± 18
HOMA index	N/A	9.9 ± 9.5
Hemoglobin, g/L	138 ± 13	140 ± 16
HCT, volume fraction	0.42 ± 0.04	0.42 ± 0.04
MCV, fL	91.3 ± 4.6	89.6 ± 3.3
MCH, pg	30.2 ± 1.9	29.9 ± 1.1
MCHC, g/L	330.7 ± 8.1	330.3 ± 16.9
Creatinine, µmol/L	79 ± 19	91 ± 29
Triglycerides, mmol/L	0.7 ± 0.1	2.0 ± 1.1***
Total cholesterol, mmol/L	3.9 ± 0.6	4.0 ± 1.2
HDL cholesterol, mmol/L	1.6 ± 0.2	1.1 ± 0.3***
LDL cholesterol, mmol/L	2.0 ± 0.5	1.9 ± 1.0
**Vascular complications, *n*** (%)		
*Microvascular complications*		
Retinopathy	8 (62)	8 (31)
Neuropathy	4 (31)	2 (8)
Nephropathy	0 (0)	4 (15)
*Macrovascular complications*		
Coronary artery disease	0 (0)	6 (23)
Peripheral vascular disease	0 (0)	1 (2)
**Medications, *n* (%)**		
*Cardiac drugs*		
ACEi/ARB	3 (23)	16 (62)*
Calcium-channel blocker	1 (8)	6 (23)
β-Blocker	1 (8)	7 (27)
*Diabetes drugs*		
Insulin	13 (100)	20 (77)
Metformin	1 (8)	15 (58)**
GLP-1 agonist	1 (8)	8 (31)
DPP-4i	0 (0)	2 (8)
SU	0 (0)	6 (23)
SGLT2i	1 (8)	1 (4)
*Other medication*		
Lipid-lowering	12 (92)	22 (85)
Aspirin	1 (8)	13 (50)*

Values are presented as mean ± SD or as number (*n*) and percent (%). BMI, body mass index; BP, blood pressure; DPP-4i, dipeptidyl peptidase-4 inhibitor; GLP-1, glucagon like peptide-1; HCT, hematocrit; HDL, high-density lipoprotein; HOMA, homeostatic model assessment; LDL, low-density lipoprotein; MCH, mean corpuscular hemoglobin; MCHC, mean corpuscular hemoglobin concentration; MCV, mean corpuscular volume; SGLT2i, sodium glucose like transporter-2 inhibitor; SU, sulfonylurea. *P<0.05, **P<0.01, ***P<0.001 vs T1DM.

### Impaired endothelial function *in vivo* in patients with T2DM compared with T1DM

**Figure 1:**
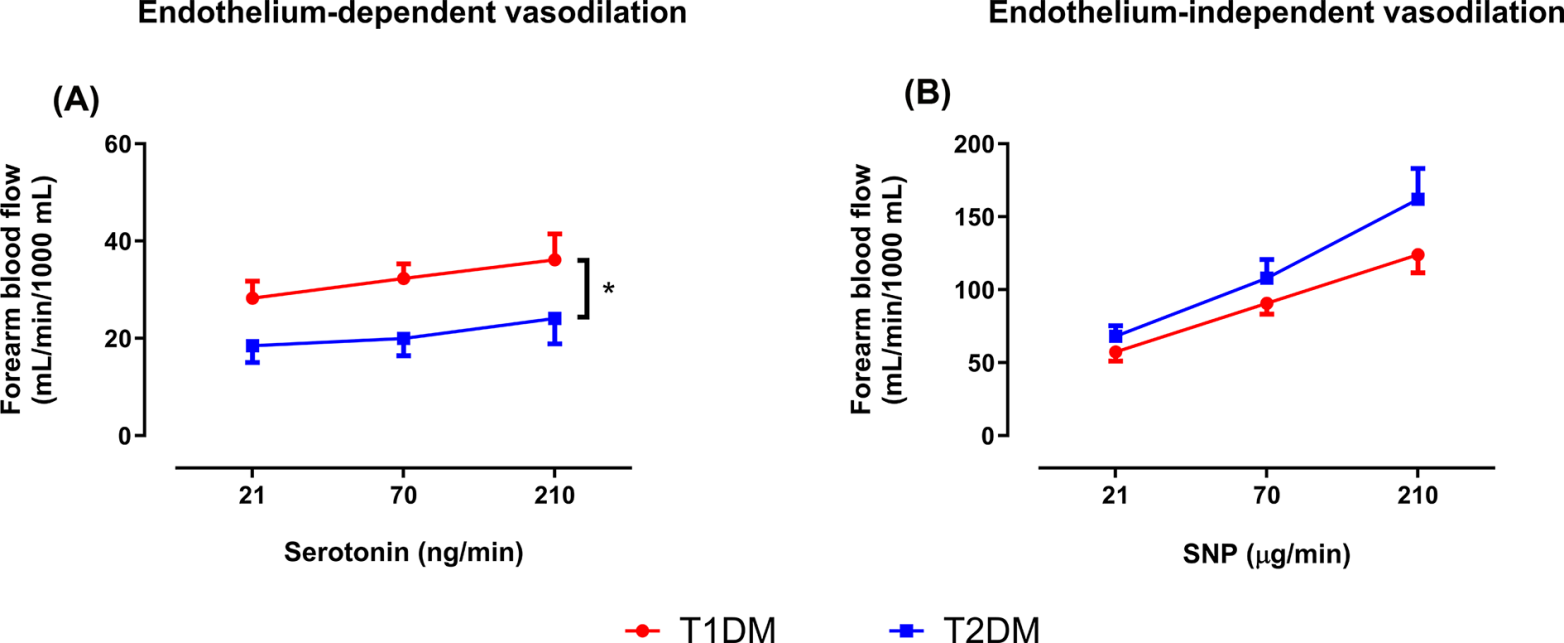
Patients with T2DM have impaired endothelial function compared with patients with T1DM Change in forearm blood flow as measures of endothelium-dependent (**A**) and -independent (**B**) vasodilatation induced by intra-arterial infusion of serotonin and sodium nitroprusside (SNP), respectively, in patients with T1DM (*n*=12) and T2DM (*n*=12). Data are presented as mean ± SEM. Statistical differences were analyzed with two-way ANOVA with repeated measures. *P<0.05 comparing the entire curves.

**Figure 2:**
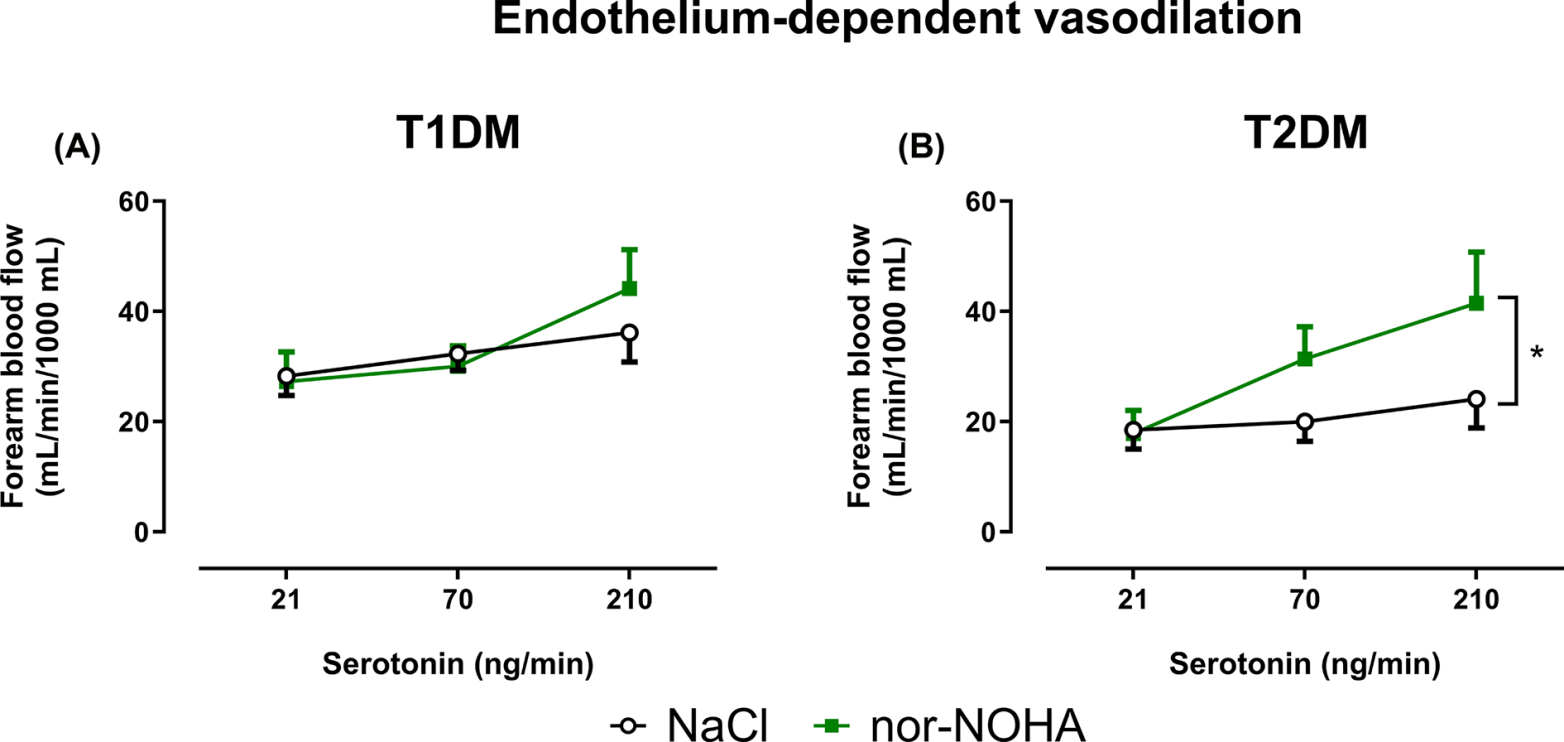
Arginase inhibition improves endothelium-dependent function in patients with T2DM but not T1DM Change in forearm blood flow as measures of endothelium-dependent vasodilatation induced by intra-arterial infusion of serotonin in patients with T1DM (**A**; *n*=12) and T2DM (**B**; *n*=12), respectively, at baseline during infusion of NaCl and after 2 h intra-arterial infusion of the arginase inhibitor nor-NOHA. Data are presented as mean ± SEM. Statistical differences were analyzed with two-way ANOVA with repeated measures. *P<0.05 comparing the entire curves.

**Figure 3:**
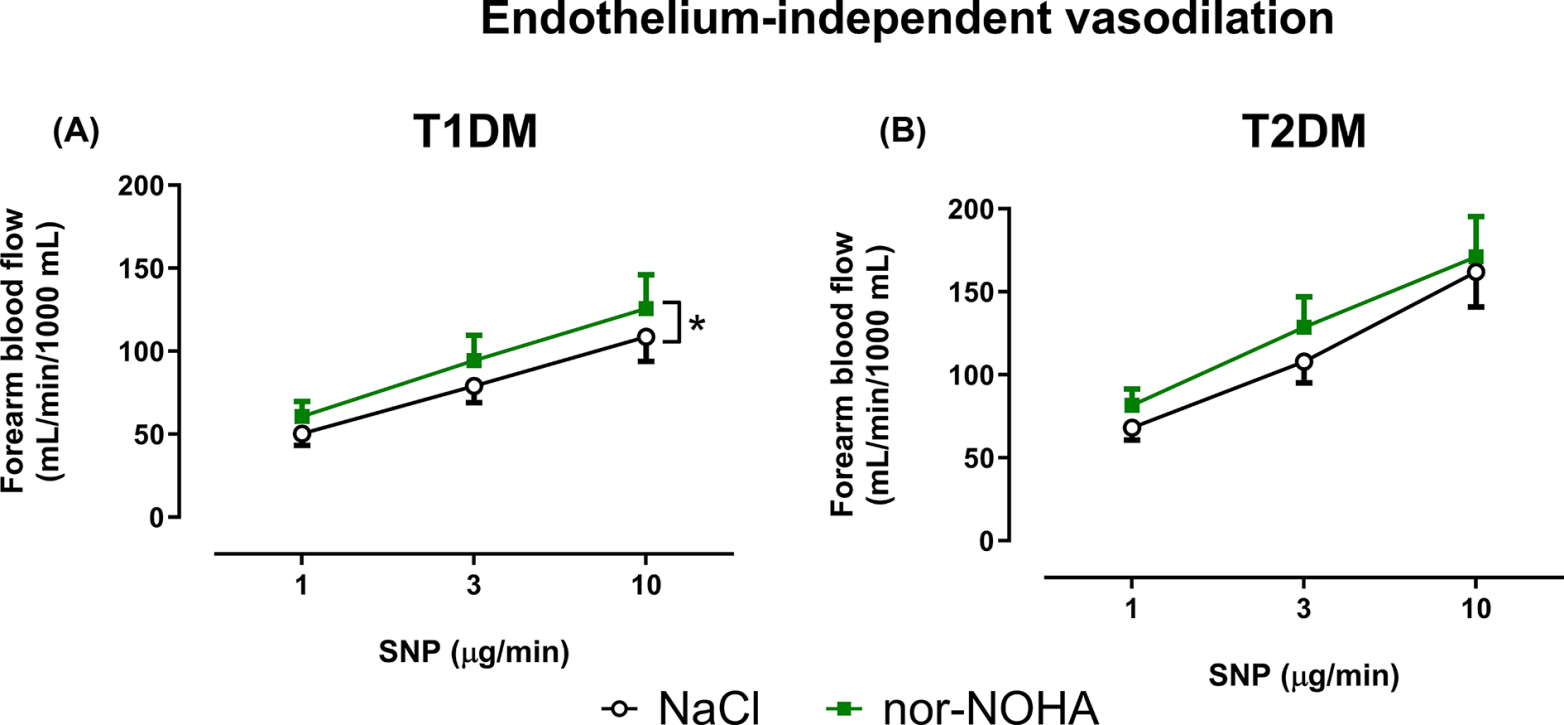
Arginase inhibition improves endothelium-independent function in patients with T1DM but not T2DM Change in forearm blood flow as measures of endothelium-independent vasodilatation induced by intra-arterial infusion of SNP in patients with T1DM (**A**; *n*=12) and T2DM (**B**; *n*=12), respectively, at baseline during infusion of NaCl and after 2 h intra-arterial infusion of the arginase inhibitor nor-NOHA. Data are presented as mean ± SEM. Statistical differences were analyzed with two-way ANOVA with repeated measures. *P<0.05 comparing the entire curves.

Baseline FBF did not differ between patients with T1DM and T2DM (Table 2). Baseline EDV (Figure 1A) was significantly lower in patients with T2DM compared with patients with T1DM, whereas EIDV (Figure 1B) did not differ significantly between the groups. Nor-NOHA did not affect baseline FBF in either group (Table 2), nor did it affect EDV in patients with T1DM (Figure 2A). By contrast, EDV was significantly improved after 2h infusion of nor-NOHA in patients with T2DM (Figure 2B). Nor-NOHA induced a slight but significant improvement of EIDV in patients with T1DM (Figure 3A) but not T2DM (Figure 3B).

**Table 2:** Forearm blood flow at baseline

	T1DM	T2DM
NaCl, ml/min/1000 ml	28 ± 14	35 ± 13
nor-NOHA, ml/min/1000 ml	32 ± 14	34 ± 20

Blood flow during infusion of NaCl, before and after 2 h infusion of the arginase inhibitor nor-NOHA.

Data are presented as mean ± SD and expressed as ml/min/1000 ml. No significant difference was observed using Mann–Whitney *U* test for comparisons between the T1DM and T2DM groups. Wilcoxon matched pairs signed rank test was used for comparisons within the T1DM group and paired t-test within the T2DM group, no significant differences were observed. nor-NOHA = N^ω^-hydroxy-nor-L-arginine.

### RBCs from patients with T2DM but not T1DM induce endothelial dysfunction

**Figure 4:**
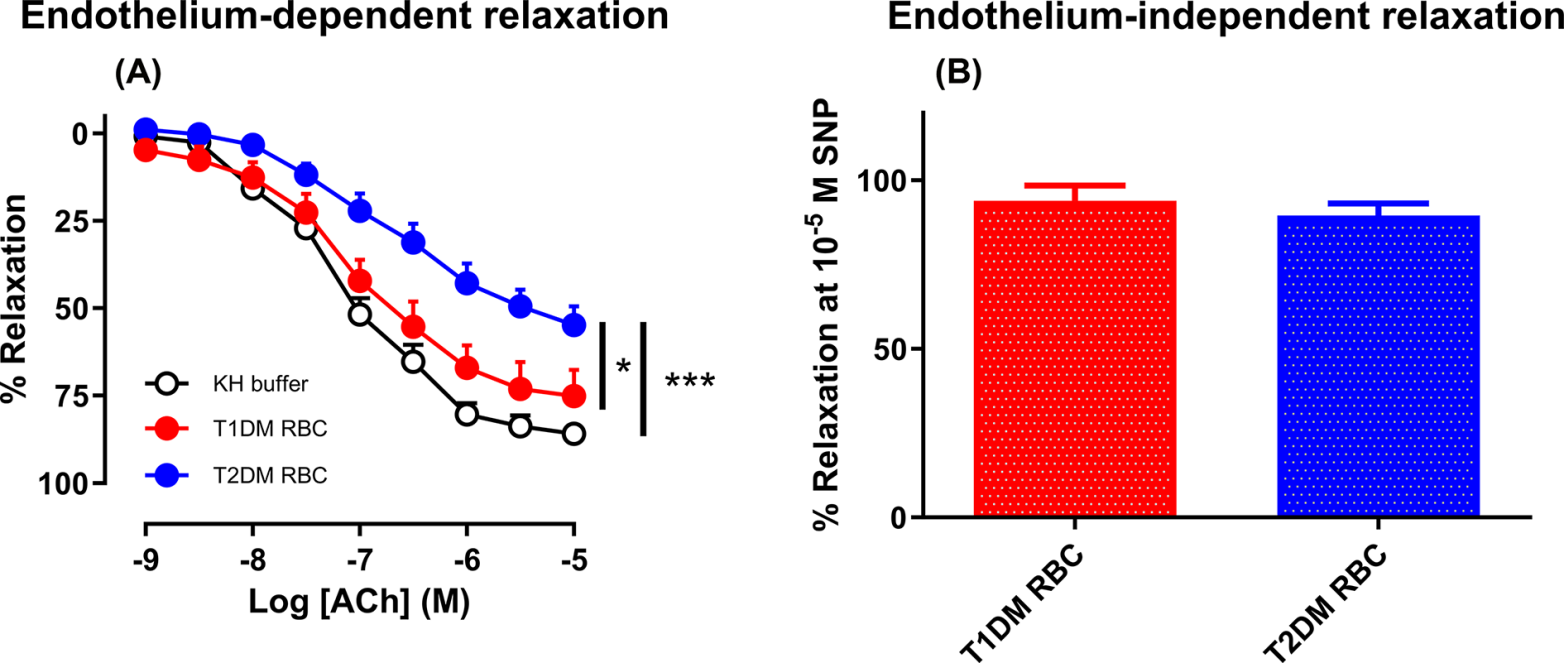
RBCs from patients with T2DM induces endothelium dependent impairment compared with RBC from patients with T1DM Endothelium-dependent relaxation (**A**) and endothelium-independent relaxation (**B**) induced by acetylcholine (ACh) and sodium nitroprusside (SNP), respectively, in isolated rat aortas after 18 h incubation with either RBCs from patients with T1DM (T1DM RBC; A; *n*=8, B; *n*=8) or T2DM (T2DM RBC; A; *n*=12, B; *n*=7) or KH buffer (KH; *n*=18) as control. Data are presented as mean ± SEM. *P<0.05, ***P<0.001 comparing the entire curves using two-way ANOVA with repeated measures (A) and Mann–Whitney *U* test (B).

We next assessed a possible mechanistic explanation behind the differences in endothelial function *in vivo* in patients with T1DM and T2DM. To this end, we focused on endothelial dysfunction induced by RBCs since this has previously been shown to be an important mediator of endothelial dysfunction in T2DM. Co-incubation of healthy rat aortas with RBCs from patients with T2DM induced impairment in EDR in comparison with aortic segments incubated in KH buffer (Figure 4A). By contrast, RBCs from patients with T1DM did not affect endothelial function in comparison with vessels incubated with KH buffer. RBCs from T1DM or T2DM patients did not affect EIDR (Figure 4B).

## Discussion

In the present study, we show that (1) patients with T2DM have markedly impaired endothelial function *in vivo* compared with patients with T1DM with comparable glycemic status, (2) arginase inhibition improves endothelial function in patients with T2DM but not T1DM and (3) RBCs from patients with T2DM but not T1DM induce endothelial dysfunction. These findings present novel insights into important differences between T1DM and T2DM regarding endothelial function, and the involvement of arginase and RBCs as mediators of this endothelial dysfunction in T2DM.

To the best of our knowledge, this is the first study comparing endothelial function in patients with T1DM and T2DM, thereby shedding new light on the differences in vascular reactivity between the two types of diabetes with comparable degree of dysglycemia. There is evidence indicating that children and adolescents with T1DM have endothelial dysfunction compared with non-diabetic patients [22–25]. However, the data from the present study performed on older patients with considerably longer disease duration, show that forearm endothelial function is clearly impaired in T2DM compared with patients with T1DM, suggesting that endothelial dysfunction could be a more important driver of vascular complications in T2DM. The degree of hyperglycemia does not explain this difference, as the patients were matched by HbA1c. This is further strengthened by the observation that improvement in glycemic status does not ameliorate endothelial dysfunction in patients with T2DM [17]. Both cohorts had a mean age over 60 and elevated HbA1c, two factors known to up-regulate the expression of arginase resulting in endothelial dysfunction [12,26]. It is unlikely that the slight difference in age between the groups explains the pronounced difference in endothelial function based on the moderate correlation between age and endothelial function among mid-aged individuals as previously demonstrated [27]. Disease duration is known to be a major determinant of endothelial function in both T1DM and T2DM [28,29]. However, this would if anything, counteract the difference in endothelial function since the patients with T1DM had a mean of 15 years longer disease duration.

As the two types of diabetes differ vastly in terms of multiple metabolic aspects, there might be several explanations, beyond hyperglycemia, for the difference in endothelial function between the two types of diabetes. Insulin resistance as a causative factor in this setting might represent one explanation based on the fact that circulating levels of insulin differ between T1DM and T2DM. Insulin resistance in T2DM, which leads to higher circulating levels of insulin, is known to play an active role in the pathophysiology of endothelial dysfunction. It has been suggested that in insulin resistance, dysregulation of the phosphatidylinositol 3-kinase pathway leads to decreased phosphorylation of eNOS and thereby decreased production of NO [30]. Subsequent insulin-dependent activation of MAP kinase in insulin resistant states leads to production of the potent vasoconstrictor endothelin-1 as well as increased expression of endothelial cell adhesion molecules [30]. The different degrees of insulin resistance and insulin levels might therefore explain the altered endothelial function in T2DM compared with T1DM. It should also be considered that the patients with T1DM and T2DM differed in terms of medication. A larger proportion of patients with T2DM were treated with metformin and inhibitors of the angiotensin system which are known to exert protective effects on endothelial function in patients with T2DM [31,32]. A majority of the patients were treated with lipid-lowering medication, mainly statins, which have been shown to protect the endothelium [33]. Also, it has been demonstrated that statins [34] and insulin [35] may decrease arginase activity. Yet, the included patients with T2DM still have prominent endothelial dysfunction despite a comparable proportion of patients with T2DM and T1DM on lipid-lowering treatment. This is in line with a previous observation, that statin treatment is not associated with attenuated RBC-induced endothelial dysfunction [19]. This could potentially be explained by that statins have modest, if any, effect on triglyceride levels and free fatty acids, which are mediators of endothelial dysfunction [36,37]. Collectively, the influence of co-medication is complex, and several of the drugs that protect from endothelial dysfunction may influence both groups of patients, and certain protective medications given to patients with T2DM may in fact underestimate the difference in endothelial dysfunction between the groups.

The role of arginase in the setting of diabetes is intriguing. Evidence clearly suggests an active role of arginase to mediate endothelial dysfunction in patients with T2DM via disruption of NO production with subsequent ROS formation due to eNOS uncoupling resulting in oxidative stress [15,16]. In experimental models of T1DM, it has been demonstrated that protein levels and activity of arginase were increased in endothelial cells, leading to endothelial dysfunction [12]. Also, hyperglycemia has been proposed to induce increased arginase activity in endothelial cells from healthy individuals [38]. Contrary to these observations, our *in vivo* data do not suggest that arginase mediate impairment in endothelial function in patients with T1DM, despite rather poor glycemic control. Furthermore, we have previously shown that improvement in glycemic status did not restore endothelial dysfunction in patients with T2DM, and the magnitude of improvement in endothelial function by arginase inhibition was similar regardless of glycemic control [17]. Potentially, hyperglycemia may act as a trigger of arginase activity in T1DM, but it may not be sufficient to sustain the increase. Unexpectedly, we observed that EIDV was slightly improved only in T1DM patients following arginase inhibition. The mechanism for this observation is unclear but may suggest altered smooth muscle cell function including altered response to exogenous NO in T1DM. To the best of our knowledge, the present study is the first to directly compare the pharmacological effect of arginase inhibition on endothelial function in patients with T1DM and T2DM *in vivo*. The present observations suggest that arginase is a key driver of endothelial dysfunction in T2DM, a feature that is not apparent among patients with T1DM. Accordingly, the present results suggest that arginase inhibition represents a promising therapeutic strategy to improve endothelial function in T2DM but not T1DM.

In an attempt to provide mechanistic insights behind the difference in *in vivo* endothelial function, we proceeded with the assessment of the role of RBCs in the two diseases. Our group has recently demonstrated that RBCs possess a pathophysiological role in T2DM by inducing endothelial dysfunction [19]. However, RBCs from patients with T1DM have not been investigated in this setting. In contrast to RBCs from patients with T2DM, our data demonstrate that RBCs from patients with T1DM do not induce endothelial dysfunction, which is in line with the *in vivo* data. Our previous results [19] show that the endothelial dysfunction induced by RBCs in T2DM is dependent on up-regulation of arginase activity and oxidative stress in the affected endothelial cells. The results from the current study showing that RBCs from patients with T1DM did not affect endothelial function indicate that RBCs do not activate these pathways in T1DM. This is supported by the *in vivo* finding that arginase inhibition did not improve endothelium-dependent vasodilatation in patients with T1DM. Although it is currently unknown which cellular compartment that is affected by administration of the arginase inhibitor *in vivo*, existing data suggest that RBCs might represent a major target to improve endothelial function. The mechanisms underlying the different effects of RBCs on endothelial function in T1DM and T2DM are unclear at present. It has been suggested that increased triglyceride levels cause increased aggregation of RBCs, possibly contributing to endothelial dysfunction through increased aggregation to the endothelium [39]. Furthermore, plasma-free fatty acids correlate to RBC lipid peroxidation, which might contribute to the dysfunction induced by RBCs in patients with T2DM [40]. Also, microRNAs, which have clear pathophysiological implications in cardiovascular disease [41], have been shown to be dysregulated in RBCs from individuals with T2DM and induce endothelial dysfunction in T2DM [42,43]. Their potentially different roles in the modulation of endothelial function in T1DM and T2DM are of interest to determine. Future studies are therefore warranted to decipher the mechanisms behind the different effects of RBCs from patients with T1DM and T2DM in more detail.

Several limitations in the current study need to be acknowledged. In the present study, we did not compare our observations to individuals without diabetes. However, a previous study demonstrated a marked attenuation of endothelial function in patients with T2DM compared with healthy subjects [15]. Interestingly, the magnitude of difference in endothelial function among patients with T1DM and T2DM in the present study is comparable to the difference in EDV observed between the patients with T2DM and healthy controls in our previous studies [15,17]. Furthermore, one should also consider that the measurements in this study are changes in forearm blood flow as measures of endothelial function which differs from other methods such as flow-mediated dilatation which measures the endothelial function in conduit arteries. The relevance of these methods for micro- and macrovascular complications may therefore vary.

In conclusion, we demonstrate differences in both *in vivo* endothelial function and RBC function between patients with T1DM and T2DM. RBCs from patients with T2DM but not T1DM induce endothelial dysfunction. Furthermore, patients with T2DM, but not those with T1DM, display improvement in *in vivo* endothelial function by arginase inhibition, indicating a therapeutic potential for arginase inhibition in patients with T2DM. Further studies are needed to delineate and dissect the exact mechanisms by which arginase and RBCs differ in T1DM and T2DM.

## Clinical perspectives

- The underlying mechanisms of endothelial dysfunction in Type 1 and Type 2 diabetes are unresolved.
- We show that endothelial function is impaired in patients with Type 2 diabetes compared with patients with Type 1 diabetes, with comparable glycemic status. Furthermore, the effect of arginase inhibition is only observed in patients with Type 2 diabetes highlighting a distinct role of arginase in Type 2 diabetes. Lastly, the red blood cell only impairs endothelial function among patients with Type 2 diabetes.
- These novel insights we present contribute to the growing body of knowledge on diabetes pathophysiology and addresses a significant gap in the current understanding of diabetes-related vascular complications.

## Data Availability

The data included in this study are available from the corresponding authors upon reasonable request.

## Open Access

Open access for this article was enabled by the participation of Karolinska Institute in an all-inclusive *Read & Publish* agreement with Portland Press and the Biochemical Society under a transformative agreement with Individual.

## Abbreviations

ACh: acetylcholine
EDR: endothelium-dependent relaxation
EDV: endothelium-dependent vasodilation
EIDR: endothelium-independent relaxation
EIDV: endothelium-independent vasodilation
eNOS: endothelial nitric oxide synthase
KH: Krebs Henseleit
NO: nitric oxide
Nor-NOHA: N^ω^-hydroxy-nor-L-arginine
RBC: red blood cell
ROS: reactive oxygen species
SNP: sodium nitroprusside
T1DM: Type 1 diabetes mellitus
T1DM RBC: red blood cell from patients with Type 1 diabetes mellitus
T2DM: Type 2 diabetes mellitus
T2DM RBC: red blood cell from patients with Type 2 diabetes mellitus
